# Current Status of Zika Virus Vaccines: Successes and Challenges

**DOI:** 10.3390/vaccines8020266

**Published:** 2020-05-31

**Authors:** Aryamav Pattnaik, Bikash R. Sahoo, Asit K. Pattnaik

**Affiliations:** 1 School of Veterinary Medicine and Biomedical Sciences, University of Nebraska-Lincoln, Lincoln, NE 68583, USA; ary@huskers.unl.edu (A.P.); bsahoo@huskers.unl.edu (B.R.S.); 2 Nebraska Center for Virology, University of Nebraska-Lincoln, Lincoln, NE 68583, USA

**Keywords:** Zika virus, vaccine platforms and vaccine candidates, correlates of protection, immunoinformatics, ADE

## Abstract

The recently emerged Zika virus (ZIKV) spread to the Americas, causing a spectrum of congenital diseases including microcephaly in newborn and Guillain-Barré syndrome (GBS) in adults. The unprecedented nature of the epidemic and serious diseases associated with the viral infections prompted the global research community to understand the immunopathogenic mechanisms of the virus and rapidly develop safe and efficacious vaccines. This has led to a number of ZIKV vaccine candidates that have shown significant promise in human clinical trials. These candidates include nucleic acid vaccines, inactivated vaccines, viral-vectored vaccines, and attenuated vaccines. Additionally, a number of vaccine candidates have been shown to protect animals in preclinical studies. However, as the epidemic has waned in the last three years, further development of the most promising vaccine candidates faces challenges in clinical efficacy trials, which is needed before a vaccine is brought to licensure. It is important that a coalition of government funding agencies and private sector companies is established to move forward with a safe and effective vaccine ready for deployment when the next ZIKV epidemic occurs.

## 1. Zika Virus: History and Biology

Zika virus (ZIKV), a mosquito-borne flavivirus, was isolated in 1947 from the blood sample of a sentinel rhesus monkey in the Zika forest of Uganda [1]. Although the first case of human infection by ZIKV was described in Nigeria in the early 1950s, only sporadic outbreaks of infections were reported in Africa and Asia over the next few decades. The virus emerged in 200,7 causing an outbreak in the Yap Island of Federated States of Micronesia, with the majority of symptomatic patients exhibiting fever, rash, and arthritis/arthralgia [2]. A larger outbreak of the virus followed in French Polynesia and other Pacific Islands in late 2013, where, in addition to the above-described symptoms, conjunctivitis was also noted [3,4]. The virus was subsequently detected for the first time in Brazil in 2015, causing infections in epidemic proportions [5], although epidemiological studies through viral genome sequence analysis suggest that the virus may have been circulating in the northeastern part of the country as early as late 2013 [6]. The virus rapidly spread to many countries in the Americas and other parts of the world [7,8]. The incidences of ZIKV infections in the Americas peaked in early 2016 with the cumulative number of documented and suspected cases exceeding 1 million. However, the number of incidences in the Americas and the world has waned significantly after 2017. 

The ZIKV epidemic in Brazil, as well as other countries, was linked to devastating congenital Zika syndrome (CZS) including microcephaly, congenital malformation, and fetal demise, particularly when women are infected with the virus during the first trimester of pregnancy [9,10,11,12,13,14,15]. ZIKV infections were also found to be associated with Guillain–Barré syndrome (GBS) in adults, an auto-immune disease of the peripheral nerves that can result in muscle weakness and paralysis [16,17,18,19]. Since ZIKV infections prior to 2013 were mostly associated with mild disease symptoms, these unexpected severe diseases such as GBS and CZS associated with the virus infections led the World Health Organization (WHO) to declare ZIKV as a Public Health Emergency of International Concern [20]. This prompted the global health and research community to not only understand the biology and pathogenesis of the virus but also devise strategies for prevention and control measures, including the development of safe and efficacious vaccines and antivirals against ZIKV. 

The primary mode of ZIKV transmission is through mosquito bite; however, the virus has been shown to be transmitted sexually [21,22,23], through the maternal–fetal route [24,25,26] and through blood transfusion [27,28]. The incubation period is estimated to range from 3 to 14 days [29]. The majority (80% or more) of individuals infected with ZIKV do not show any symptoms while some develop mild symptoms including fever, rash, conjunctivitis, headache, malaise, muscle, and joint pain lasting for 2 to 7 days, that self-resolve with time. However, in a very small number of cases, infections can lead severe diseases, such as GBS in adults and CZS in pregnant women, with a variety of pathological abnormalities of the fetus such as craniofacial, musculoskeletal, ocular, pulmonary, in addition to microcephaly [14,30]. Recent studies have revealed that ZIKV infection of male mice can result in virus persistence and testis damage leading to reduced fertility [31,32]. In addition, case studies in Brazil have also suggested that ZIKV infection may be responsible for reduced fertility in men [33]. Zika virus infection also results in conjunctivitis and causes persistent chorioretinal lesions with uveitis in humans and mice models [34,35]. Unlike the previous ZIKV outbreaks, it is still not fully understood as to which genomic changes may have resulted in the high pathogenicity of the virus in recent outbreaks with such a wide spectrum of diseases. 

Serologic and genome analyses suggest the existence of only one single serotype with three distinct genetic lineages: East African (that includes the first isolate from Uganda, MR766), West African, and Asian, including the first isolate from Asia (P6-740) and all contemporary strains from Asia, Oceania, and the Americas [36,37]. Like most other mosquito-transmitted flaviviruses [such as dengue virus (DENV), West Nile virus (WNV), Japanese encephalitis virus (JEV), yellow fever virus (YFV)], ZIKV is enveloped and contains a positive-sense, single-stranded RNA genome of ~11 kilobases. The genome encodes a single ORF, which is translated to produce a large polyprotein that is processed by viral and cellular proteases leading to three amino–terminal structural proteins (capsid, C; pre-membrane, prM; and envelope, E) and seven carboxy–terminal non-structural (NS) proteins (NS1, NS2A, NS2B, NS3, NS4A, NS4B, and NS5). While the structural proteins are primarily involved in virion assembly, attachment, and entry into host cells, the NS proteins participate in viral genome replication, virion assembly, polyprotein processing, and evasion of host antiviral responses. The virions are assembled in the endoplasmic reticulum as immature virions containing 60 copies of the prM-E heterotrimers. During transport through the secretory pathway, prM molecules are cleaved by the host furin-like proteases in the trans-Golgi region into pr and M polypeptides, but following the release of the virions from the host cell, the pr peptide is removed, and M-E are rearranged into 90 copies of heterodimers generating thermally stable infectious virions [38,39,40]. Like most other flavivirus E proteins, the ZIKV E protein elicits neutralizing antibody responses [41], and therefore has been the major target for development of vaccines. Other viral proteins such as prM and NS1 proteins have also been used as targets for vaccine development.

## 2. Immune Reponses to Zika Virus Infection, Vaccination, and Correlates of Protection

Zika virus induces both innate and adaptive immune responses by the host. It is well established that type I interferon (IFN) induction and signaling are able to restrict ZIKV infection in cell culture and in mouse models [42,43,44]. On the other hand, ZIKV inhibits IFN induction and signaling by a variety of mechanisms [45]. All of the viral NS proteins (NS1, NS2A, NS2B, NS3, NS4A, NS4B and NS5) appear to play role(s) in the inhibition of type I IFN production and IFN-stimulated gene (ISG) induction [45]. The observations that ZIKV replicates well and causes disease in animals lacking components of innate immune signaling pathways such as IFNα/β receptor [44], STAT2 [46] suggest that innate immune response to ZIKV infection is the first line of defense against the pathogen. 

The adaptive immune responses (humoral and cellular) to ZIKV are protective. The humoral responses generating virus-specific neutralizing antibodies predominantly directed against the viral E protein appear to be most important for protection [47,48,49]. Overall, these antibodies fall into several classes based on their ability to recognize different regions or structures of the E protein. They either recognize a quaternary epitope on the E protein dimer–dimer interface and protect the vertical transmission of ZIKV in mice model [47,50]; recognize residues in the lateral ridge epitope of domain III (DIII) and block ZIKV infection at a post-attachment stage [51]; or recognize a quaternary epitope spanning all three domains of the E dimer, protecting mice from lethal virus challenge and vertical transmission [52,53]. This last class of antibodies also cross-reacts with DENV and enhance its replication [48] with implications for the antibody-dependent enhancement (ADE) of infection and disease. Therefore, the design of an ideal vaccine that induces antibody responses should take ADE into consideration. 

Although humoral responses to ZIKV infections have been well characterized and documented, cellular immune responses are less well studied. ZIKV-specific CD4+ and CD8+ T cell responses directed at the C, E, and the NS1 proteins have been demonstrated in virus-infected humans, non-human primates (NHPs), and mice [48,54,55,56,57,58]. A protective role for CD8+ T cells against ZIKV was demonstrated by showing that (i) CD8-/- mice display higher virus replication and increased mortality [59], and (ii) adoptive transfer of ZIKV-immune CD8+ T cells reduces viral burden whereas their depletion leads to increased viral burden [60]. Although neutralizing antibodies and CD8+ T cells are primarily responsible for the protective adaptive immune response to ZIKV, the role of CD4+ T cells is less clear. It appears that CD4+ T cells with Th1 phenotype and IFNγ signaling are required for neutralizing antibody response [58,61,62,63] and thus may play an indirect role in protection from ZIKV. An optimal vaccine candidate, therefore, should have the ability to induce both CD4+ and CD8+ T cell responses in addition to strong humoral responses. 

Early studies demonstrating that infection of rhesus macaques with ZIKV could induce adaptive immune responses including neutralizing antibody production and protect the animals from challenge with the homologous virus suggest that adaptive immune responses are protective [56]. However, defining the immune correlates that are associated with protection from infection and/or disease is challenging, since (i) different vaccine candidates induce different degrees of B and T cell responses, (ii) the immune correlates of protection will be different for different human target populations such as children, adults, and the fetus, in addition to individuals with pre-existing flavivirus immunity, with neurologic disorders, autoimmune diseases, immunosuppression, women of reproductive age, pregnant women, etc., (iii) correlates of protection in humans would likely be different from those obtained from various animal models, and (iv) human vaccine clinical trials would face considerable ethical issues, particularly with inclusion of pregnant women, given the as-yet fully uncharacterized spectrum of congenital diseases that may be associated with the virus infection. 

Despite these challenges and shortcomings, multiple ZIKV vaccine candidates under various platforms have been developed over the last four years to determine if vaccines can confer protection against the virus challenge in animal models [64,65,66,67]. These include inactivated vaccines, live attenuated vaccines, and subunit vaccines. Many of these vaccine candidates induce high levels of neutralizing antibodies as well as cellular immune responses that confer protection in mice and NHP models. The general consensus from these studies is that protective efficacy of a vaccine is strongly linked to neutralizing antibody response, although in one study, protection was found to be independent of neutralizing antibodies in a phase I clinical trial [68], where the adoptive transfer of sera containing non-neutralizing, ZIKV-binding antibodies from several DNA-vaccinated humans was shown to protect mice by an as-yet undetermined mechanism. The serum antibody titers required for the protection of mice and NHPs in challenge studies have been estimated to be approximately 100 to 1000 [69,70,71]. Although the protective efficacy of many of the vaccine candidates has been tested in mice and NHPs, the serum titers of the neutralizing antibodies required for protection in humans will likely be different and must be determined through clinical trials. Using purified formalin-inactivated Zika virus vaccine (ZPIV) or DNA vaccine in human clinical trials, antibody titers of ≥10 and as high as over 100 have been generated [72,73]. Based on previously licensed flavivirus vaccines such as those for JEV, YFV, etc., neutralization titers of 10 have been considered as surrogates of protection [74]. Therefore, it is conceivable that the antibody titers of greater than 10 might be adequate for ZIKV protection in humans, although the precise titers for complete protection needs to be determined from further clinical studies. Furthermore, data from randomized placebo-controlled clinical trials in volunteers from ZIKV and other flavivirus endemic areas are needed before any vaccine for ZIKV is deployed. Additional considerations must also be given to establish correlates of protection in pregnant women and the fetus from congenital malformation. 

## 3. Antibody-Dependent Enhancement (ADE) of Infection and Disease: Implications for ZIKV Vaccine Design 

ZIKV, DENV, as well as other flaviviruses, share considerable genetic and structural similarities [75] and therefore generate cross-reactive antibodies [76,77] that can be poorly neutralizing but can potentially cause antibody-dependent enhancement (ADE) of virus infection and disease. It has been found that cross-reactive DENV antibodies that are poorly neutralizing or neutralizing antibodies at subneutralizing concentrations can promote virus entry into cells bearing FcγRs, enhance virus replication, and exacerbate the virus associated diseases in humans [78,79,80]. Epidemiological studies suggest that previous infection with a DENV serotype may predispose an individual to more severe disease such as hemorrhagic fever due to secondary infection with an unrelated serotype of the virus [81]. The secondary infection in the presence of these antibodies enhances virus uptake and replication, which may then result in a cytokine storm through the activation of memory T cell responses, leading to the enhancement of the disease. Since ZIKV infections occurred in many places where DENV is endemic, it was postulated that the presence of previous DENV antibodies may have exacerbated ZIKV infections and related diseases. In fact, studies show that human antibody responses following DENV or other flavivirus infections are highly cross-reactive to ZIKV [82] and that such antibodies mediate the ADE of ZIKV infection in cell culture and mice, although this has not been seen in NHPs [83,84,85]. Likewise, cross-reactive ZIKV antibodies have been shown to mediate ADE of DENV [48,86]. Whether pre-existing antibodies to DENV mediate the ADE of ZIKV in humans is not clear at this time, but a preliminary epidemiological study has found no evidence for this [87]. Interestingly, a recent study [88] demonstrated that the administration of a ZIKV inactivated vaccine in DENV-experienced humans resulted in potent cross-neutralizing antibodies to both ZIKV and DENV, suggesting that ZIKV vaccination in DENV and other flavivirus-endemic areas might be beneficial.

Examination of human monoclonal antibodies from flavivirus-infected individuals suggests that the fusion loop epitope (FLE) within the E protein domain II (EDII) is an immunodominant epitope with broad cross-reactivity to flaviviruses [76,77,78]. The FLE-specific antibodies directed against DENV- and ZIKV-infected individuals have been shown to induce the ADE of ZIKV in vitro and mice model [83,89]. Another immunodominant epitope within the prM has also been identified that generates cross-reactive antibodies and induces ADE [76,77,78]. Therefore, ZIKV vaccine design approaches should consider abrogating ADE. This could be achieved by using E protein regions that lack FLE or having immunogens where the exposure of FLE to the host’s immune system is blocked or minimized. Other vaccine candidates that circumvent the risk of ADE are also being developed. Notable among those is the NS1 protein, which has been shown to induce protective antibody responses as well as T cell responses [63,90,91,92]. Although it is typically not considered an antigen for vaccine development, flavivirus NS1 is immunogenic, expressed intracellularly and on the surface of infected cells as well as in secreted form, and is shown to induce protective immune responses [93,94,95].

## 4. Zika Virus Vaccine Development: Current Status

**Figure 1:**
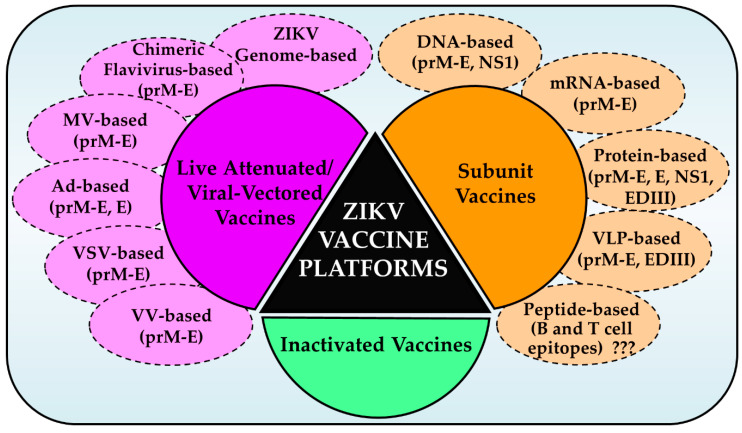
Schematic of various ZIKV vaccine platforms. Three major platforms: inactivated, subunit, and live attenuated/viral-vectored vaccines have been used to generate a number of vaccine candidates for preclinical and clinical testing. A variety of approaches and immunogens as shown were used. Immunoinformatics-based peptide vaccines have not been generated/tested (???). MV, measles virus; Ad, adenovirus; VSV, vesicular stomatitis virus; VV, vaccinia virus.

With the explosion of ZIKV cases in the Americas in 2016 and established association with neurological and congenital diseases, intense efforts were made towards the development of a number of vaccine candidates that could be tested in preclinical and clinical trials. The WHO ZIKV vaccine development roadmap provided a framework of two scenarios for such vaccine use: outbreak use, and endemic use [96]. The former scenario entails the mass vaccination of susceptible population including pregnant women and women of child-bearing age during an ongoing epidemic while the latter involves the broad vaccination of the general population during inter-epidemic times. The accelerated development of several ZIKV vaccine candidates was largely due to past experience with the development of several successful flavivirus vaccines and more detailed knowledge of the biology and pathogenesis of ZIKV and development of animal models [97]. Several different ZIKV vaccine candidates under different platforms (Figure 1) have now been developed and tested in preclinical and clinical trials. These include: nucleic acid vaccines (DNA and RNA vaccines), inactivated whole virus vaccines, live attenuated vaccines, viral vectored vaccines, protein antigen vaccines in the form of purified proteins from expression systems, or virus-like particles. Several of these candidates are in various stages of preclinical studies or in human clinical trials. Although the development of peptide vaccines based on immunoinformatics approaches has been proposed, such vaccines have not come to a stage for preclinical trials yet.

Each of these vaccine platforms induce humoral and/or cell-mediated immune responses to varying degrees. The inactivated vaccines are usually safe and could be administered to most target populations including pregnant women as well as women of child-bearing ages. However, multiple doses and use of adjuvants may be needed for the induction of more robust and long-lasting protection. ADE is also a concern with these vaccines. Among subunit vaccines, nucleic acid-based vaccines using prM-E genes generate VLPs similar to virus particles that are released from cells and therefore these, along with VLP-based vaccines, present the viral antigens to the host immune system, similar to the infectious virus. Additionally, some subunit vaccines based on NS1 or EDIII are designed to avoid ADE. The subunit vaccines are generally considered as safe, depending the nature of the adjuvants, for use in all target population, including pregnant women. Live attenuated and/or several of the viral-vectored vaccines also generate infectious particles and/or VLPs and have similar advantages to some of the subunit vaccines. However, these vaccines, along with live attenuated ZIKV vaccines, could present problems with potential reversion to virulent forms, could induce toxic inflammatory responses, or could be less effective due to pre-existing immunity to the vectored virus.

### 4.1. Nucleic Acid Vaccines 

Nucleic acid vaccines have been under development as potential vaccine candidates for over 25 years as they can be generated fairly quickly from the genetic sequence of the desired antigen or protein. Additionally, these vaccine candidates bypass the cumbersome process of expressing the antigen or protein and having it purified prior to use. Therefore, both DNA and mRNA vaccines have generated significant interest as vaccine candidates in recent years [98,99]. However, the potential disadvantages of nucleic acid vaccines include relative instability, delivery, integration into the host genome, toxicity, and immunostimulatory and inflammatory responses by the host. Notwithstanding these limitations, nucleic acid vaccines have become the choice of future vaccine platforms by mitigating the limitations and increasing the potency of these platforms [100]. Several DNA and RNA vaccines candidates for ZIKV have now been developed and are in various stages of human clinical trials.

#### 4.1.1. DNA Vaccines

The proof-of-concept for use of a DNA vaccine against ZIKV was first developed using the coding sequences of the viral prM-E regions in plasmids and examining immune responses in mice [101] and NHPs [70]. In these studies, the plasmid constructs generated prM-E or M-E (with deletion of pr region) proteins. The induction of high levels of ZIKV-specific neutralizing antibodies was observed when the plasmid DNA was injected into mice or NHPs [70,101]. In a similar approach, two other DNA constructs were generated based on prM-E sequences as well [69]. JEV signal sequences (SS) were inserted in the place of ZIKV prM SS at the amino-terminal region in the plasmid VRC5283 whereas in the plasmid VRC5288, sequences encoding the carboxy-terminal 98 amino acids of E were replaced with the corresponding sequences from JEV in addition to the JEV SS at the amino-terminus. These constructs directed the expression of prM-E and secretion of subviral particles (SVPs) with VRC5288 directing significantly higher levels of secretion of SVPs into culture supernatants. Rhesus macaques vaccinated with these DNA constructs elicited a robust neutralizing response that protected the animals from ZIKV challenge. The VRC5283 vaccine candidate exhibited better protection, likely due to superior immunogenicity of the encoded protein. Additionally, the VRC5283 vaccine was also shown recently to protect immunocompromised mice during pregnancy and against vertical transmission and fetal demise [102]. Both of these constructs were used in phase I human clinical trials, with VRC5283 advancing to phase II clinical trials (Table 1). 

In a separate study, a consensus sequence of prM-E was generated from ZIKV strains isolated between the years 1952 and 2015 and optimized for expression and secretion with the addition of the SS of immunoglobulin [103] and cloned in a DNA vaccine vector. Following enhanced DNA delivery by electroporation using Inovio’s proprietary intradermal DNA delivery device, the vaccinated animals generated neutralizing antibodies and antigen-specific cellular immune responses that protected mice and NHPs from viremia and testes damage upon virus challenge [104]. This vaccine, GLS-5700, in phase I clinical trials, elicited ZIKV-binding antibodies in 100% individuals but only 60% of individuals in the study had neutralizing antibodies [68]. However, the immune responses were found to be protective in in vitro and in vivo models of ZIKV infection. Other DNA vaccine candidates that have demonstrated promise in preclinical/animal studies include NS1 as the target. A DNA vaccine encoding a secreted form of NS1 protein was shown to protect immunocompetent mice from systemic ZIKV infection. Although the NS1-specific antibodies failed to protect the mice from the virus infection, functional NS1-specific T cell responses were critical for protection [63]. On the other hand, another NS1-expressing DNA vaccine in the presence of two boosters of immunization with soluble NS1 was shown to induce high titers of antibody that, upon passive transfer, could protect immunocompromised mice against ZIKV lethal challenge [91]. Interestingly, these NS1-specific antibodies are long-lasting and were responsible for Fc-mediated effector functions for protection [91].

#### 4.1.2. mRNA Vaccines

Due to the inherent instability of mRNA and its ability to activate innate immune signaling pathways, use of modified nucleosides during in vitro transcription was shown to render mRNAs less immunogenic [105] and also enhanced its translational capacity [106,107]. In vitro transcribed mRNAs incorporating 1-methylpseudouridine in place of uridine and/or 5-methylcytidine in place of cytidine have now been used as vaccine candidates for infectious diseases and cancer [99]. Using this approach, several ZIKV RNA vaccine candidates [71,102,108,109] have been developed and evaluated in preclinical and human clinical trials. 

An mRNA for the prM-E region of the ZIKV H/PF/2013 was synthesized in vitro in the presence of the modified nucleoside, 1-methylpseudouridine and encapsulated with lipid nanoparticles (LNP). In immunocompetent mice and rhesus macaques, single intradermal injection of the nucleoside-modified mRNA-LNP induced a robust neutralizing antibody response and ZIKV-specific cellular responses that conferred complete protection form the virus challenge [71]. In a similar approach, another group also developed prM-E-expressing mRNA vaccines [108] that could protect mice from lethal ZIKV challenge and confer sterilizing immunity. A prime-boost approach was found to induce very high levels of neutralizing antibody titers. In follow up studies, these mRNA vaccines were shown to mediate protection against ZIKV virus-induced congenital disease [109] and protect immunocompetent and immunocompromised mice during pregnancy [102]. These latter mRNA vaccines (mRNA-1325 and mRNA-1895) are undergoing phase I clinical trials (Table 1) by Moderna Therapeutics, a Cambridge, MA based Biotech Company.

### 4.2. Live Attenuated Vaccines 

With the development of infectious molecular clones of many wild-type and vaccine strains of flaviviruses, it has been possible to generate live attenuated vaccine candidates through genetic manipulation. At least two major strategies have been employed to develop live attenuated ZIKV vaccines: (i) introducing specific attenuating mutations into ZIKV genome, and (ii) generating chimeric flaviviruses expressing the prM-E genes of ZIKV in the genetic background of DENV, JEV, or YFV. Through the introduction of 10 or 20 nucleotide deletions within the 3′-untranslated region (UTR) of the Cambodian strain (FSS13025) ZIKV, the deletion mutant viruses were found to be highly attenuated, and conferred sterilizing immunity in immunocompromised mice with high titers of neutralizing antibodies and robust T cell responses [110]. Additionally, while the 3′-UTRΔ10 vaccine candidate prevented the vertical transmission of the challenge virus in pregnant mice, both 3′-UTRΔ10 as well as 3′-UTRΔ20 vaccine candidates were shown to prevent testis damage and injury in male mice [111]. In NHPs, a single-dose of both vaccines induced immune response to block viremia while the 3′-UTRΔ20 virus elicited sterilizing immunity [111]. In addition, by eliminating glycosylation sites in E and/or NS1 proteins, it was also shown that the mutant viruses were attenuated, induced robust neutralizing antibody responses as well as T cell responses that in immunocompromised mice, conferred protection [112,113]. 

Chimeric DENV serotype 2 and JEV-encoding prM-E proteins of ZIKV, in place of their corresponding proteins, have also been generated [114,115]. While the DENV-2/ZIKV chimeric vaccine conferred protection of immunocompromised mice [114], the JEV/ZIKV chimeric vaccine protected mice and NHPs from ZIKV challenge as well as from placental and fetal damage [115]. Perhaps the most interesting live attenuated chimeric Zika virus vaccine candidate that has entered phase I clinical trials is the chimeric vaccine in the genomic backbone of DENV serotype 4 that expresses the prM-E proteins of ZIKV. This vaccine, rZIKV/D4Δ30-713, contains a 30-nucleotide deletion in the 3′-UTR of the DENV genome that results in reduced replication and significant attenuation in NHPs and humans. One of the major challenges for an efficacious ZIKV chimeric vaccine using DENV or JEV as genomic backbones is the presence of pre-existing immunity against these flaviviruses. Since there are cross-reactive antibodies in flavivirus endemic areas, it is possible that these antibodies could inhibit or alter the immune responses elicited by a flavivirus-based chimeric ZIKV vaccine. Response to such chimeric ZIKV vaccines might be different in individuals with pre-existing flavivirus antibodies as compared to those without. Additionally, as discussed above, such pre-existing antibodies could also enhance vaccine-mediated pathogenicity, possibly via ADE. Furthermore, it should be borne in mind that chimeric flavivirus-based ZIKV vaccine candidates could potentially pose risks of exacerbated disease in flavivirus naïve population as has been observed with the chimeric attenuated yellow fever virus (17D) genome-based tetravalent DENV vaccine [116], where administration of the vaccine (Dengvaxia^®^) to naïve children resulted in more severe Dengue-related complications as compared to the unvaccinated group [117].

It should be noted that live attenuated ZIKV vaccines or live viral-vectored vaccines could present potential safety concerns for pregnant women if these vaccines breach the maternal–fetal barrier and cause fetal damage. These vaccines should be carefully evaluated for their safety with more stringent data on maternal transmission using various animal models including NHPs, even though there is no certainty that these preclinical data will be reproducible in humans. Therefore, most vaccine developers prefer the administration of inactivated or subunit vaccines without adjuvants, or with adjuvants that are currently approved for use in other vaccines for pregnant women.

### 4.3. Vaccines Employing Other Recombinant Viral Vectors

Replication-competent or -defective viral vectors expressing the prM-E genes of ZIKV have also been developed as vaccine candidates. These include vesicular stomatitis virus (VSV), measles virus (MV), vaccinia virus, and adenovirus (Ad). An attenuated version of a recombinant VSV expressing the prM-E of ZIKV elicited both neutralizing antibody responses and T cell responses that protected challenged newborn mice born to vaccinated female mice [118]. A live attenuated measles virus vector expressing prM and soluble E (MV-Zika-sE) was shown to protect mice from ZIKV challenge through the development of E-specific neutralizing antibodies and cellular immune responses [119]. The immunization of NHPs also resulted in Zika-virus-specific neutralizing antibody responses in all vaccinated animals. This vaccine and another similarly developed MV vaccine (MV-ZIKV-RSP) are in human phase I clinical trials (Table 1). A vaccinia-based single vector construct expressing prM-E of ZIKV and the structural proteins of chikungunya virus (C-E3-E2-6K-E1) induced neutralizing antibody responses to both viruses in immunocompetent and immunocompromised mice and blocked ZIKV viremia and disease [120]. Additionally, this vaccine candidate also blocked the vertical transmission of ZIKV in immunocompromised female mice and testes damage in male mice [120]. Several adenovirus vectors expressing prM-E and/or E alone have been developed and shown promise as potential vaccines for ZIKV [70,101,121,122,123,124,125]. These vectors encode prM-E or E alone and have been shown to induce neutralizing antibodies and cellular immune responses that confer protection in mice and NHPs. The protection in NHPs has been found to last for at least one year [121]. Interestingly, one adenovirus-based vector only induced a T cell-biased protective response without neutralizing antibody response [124]. A vaccine candidate based on chimpanzee adenovirus ChAdOx1 encoding prM-E (without the transmembrane domain) of ZIKV is currently in phase I clinical trials (Table 1). 

### 4.4. Virus-Like Particle (VLP) Vaccines and Subunit Protein Vaccines

Virus-like particles (VLPs) containing the structural proteins of ZIKV, namely, prM-E have been generated in various expression systems and examined for their ability to induce neutralizing antibody response as well as cellular immune responses that can protect from the virus challenge. The DNA- and RNA-based vaccines and recombinant viral vectors described above generated VLPs directly in the animals for the induction of a protective immune response. However, the use of various expression systems to generate and purify the VLPs for administration into animals has advantages in that the animals respond to only the viral antigens without any other components. Several such vaccine candidates have been tested in preclinical studies and shown to induce neutralizing antibody and cellular immune responses [126,127,128,129] and protect immunocompromised mice against lethal virus challenge [128]. 

Purified recombinant ZIKV proteins have also been developed as vaccine candidates, although their utility in clinical studies has lagged compared to other vaccine candidates. These subunit protein vaccines are considered to be potentially safe in most target populations but have limitations in that they are not as immunogenic as the other vaccines, due to the fact that they are likely not presented to the host immune system in their native conformation. However, modifications to protein structures, particularly through the introduction of mutations to stabilize the quaternary structures of the viral E protein, have resulted in vaccine candidates that, in immunized mice, induce neutralizing antibody responses and confer protection against ZIKV challenge [130,131] during pregnancy in addition to eliminating ADE [130]. 

In an attempt to avoid ADE, others have developed vaccine candidates by expressing only the domain III (EDIII) of the E protein. E. coli expressed EDIII elicited neutralizing antibody titers in immunocompetent mice that could neutralize ZIKV in vitro and did not exhibit ADE [132]. In a separate study, a non-neutralizing epitope in EDIII was shielded by the introduction of a glycosylation site at residue 375. The modified EDIII protein induced significantly higher neutralizing antibodies in mice and fully protected pregnant mice and their fetus against lethal challenge [133]. When EDIII was presented on the surface through chemical crosslinking with an immunologically optimized VLP generated from cucumber mosaic virus with a Th cell epitope from tetanus toxin (CuMVtt), the resulting VLP induced neutralizing antibodies with no ADE activity [134].

## 5. Immunoinformatics Approach for Peptide Vaccines

As described above, numerous vaccine candidates for ZIKV have entered preclinical and clinical evaluations. The antigens for these vaccine candidates have often been comprised of the full-length or specific domains of the viral proteins. In recent years, the ability to predict B cell and T cell epitopes using a variety of bioinformatics and immunoinformatics tools [135] coupled with the availability of homology modeling and molecular docking methods, have added a new dimension to vaccinology: the peptide vaccines. The peptide vaccine approach has gained interest since a single peptide incorporating multiple dominant B cell and T cell epitopes from a number of viral proteins can be designed and used as an immunogen. These computational methods have been applied to in silico prediction for the development of ZIKV peptide vaccines [136,137,138,139]. Both linear and conformational epitopes for B and T cell responses have been predicted for the entire ZIKV proteome that could be incorporated in the design of peptide vaccines. However, it should be noted that these peptide vaccines have not been tested yet for their ability to induce a protective immune response to ZIKV infections. In one study [139], a multi-epitope-based-peptide vaccine immunogen was designed so that it contained multiple immunodominant epitopes from the ZIKV proteome. This immunogen, a 435-amino-acid-residues-long protein, contains multiple epitopes tandemly pieced together, with linkers separating the epitopes and an adjuvant at the amino-terminus. It would be interesting to examine whether such an in silico generated immunogen would induce the predicted humoral and cell-mediated immune responses for protection against ZIKV.

## 6. Challenges and Future Perspectives

Over the last four years, significant efforts have been made by researchers worldwide to develop safe and efficacious vaccines for ZIKV. This has resulted in the development of at least 13 vaccine candidates under various platforms that have entered 17 different human phase I clinical trials with one entering phase II clinical trial. Additionally, many vaccine candidates have been tested in preclinical studies and have demonstrated significant promise for further development. Undoubtedly, this is considered a major success, although much work remains to be completed to bring a ZIKV vaccine to licensure and for public use. Several concerns need to be addressed before a successful ZIKV is deployed.

1. Since cases of human ZIKV infections have essentially disappeared in the past two years, efficacy testing of ZIKV vaccine candidates in target populations is challenging. Alternatively, a controlled human infection model could be developed not only to test vaccine efficacy but also determine the correlates of protection in humans. The development of such models needs the participation of volunteers that require approval by appropriate regulatory bodies, which should take into consideration the ethical issues as well as social benefits of infecting the volunteers with ZIKV. Given this situation, the WHO, and the NIH (USA) concluded that ZIKV vaccine efficacy trials in the absence of outbreaks are not feasible, and that a robust definition of immunological markers predicting protection should be developed [140] before a ZIKV vaccine can move to licensure;
2. A sustained source of support for the development of vaccines should be identified/maintained in the face of a waning epidemic. This has been a serious concern with ZIKV vaccine development projects. When a sudden and significant outbreak of an epidemic occurs that threatens public health, emergency funding from governments becomes readily available, only to dry out later as the epidemic wanes, leaving the vaccine development projects incomplete. Although the US Food and Drug Administration (FDA) has instituted incentive programs for developing vaccines that are not profitable for developers, a more robust partnership between government and private sectors should be built to address these concerns;
3. Recent studies suggest that pre-existing flavivirus antibodies in humans may not be a major concern for the ADE of ZIKV; however, more detailed investigations are needed. Likewise, studies should also address whether administering ZIKV vaccines to naïve or flavivirus-exposed individuals would affect the efficacy of the vaccine;
4. Since ZIKV causes a variety of congenital diseases and GBS, whether a single vaccine would adequately protect pregnant women, women of child-bearing ages, children, and adults need to be determined. Several vaccine candidates have been shown to protect the placenta and the fetus of pregnant mice and NHPs and testis damage in males from ZIKV; whether these vaccines can protect pregnant women and the unborn remains to be determined. What are the correlates of protection in these target populations? It is likely that different types of vaccines are needed for different target populations, as the correlates of protection may be different for each group and vaccine type. This should be determined with additional studies. Answers to these critical questions through further preclinical studies and clinical trials would help in developing an effective vaccine.

## 7. Summary and Conclusions

Since the declaration of ZIKV and its associated diseases as a public health emergency, remarkable progress has been achieved for the development of ZIKV vaccine candidates. Many of these candidates have shown significant promise in various animal models and several have now entered human clinical phase I/II trials. However, further evaluation of these vaccine candidates in human clinical trials appears unlikely, due primarily to lack of funding, the high financial costs associated with these studies and uncertain levels of profit for the private sector companies. Partnerships between government institutions and private sector companies should enable further clinical testing and eventual licensure and manufacturing of ZIKV vaccines ready for deployment, should a ZIKV epidemic occur in the future. As ZIKV has caused a significant epidemic in the Americas and many other parts of the world recently, it is uncertain when and where the next epidemic will occur. The scientists, biopharmaceutical companies, public health officials, and policy makers must come together to prepare well for the next outbreak of ZIKV with efficacious vaccines and antivirals.

## Figures and Tables

**Table 1:** ZIKV vaccine candidates and their status in clinical trials.

Platform	Vaccine Name	Sponsor	Antigen	Status in Clinical Trials	
				Phase 1	Phase 2
DNA	VRC5283	NIAID/VRC	prM-E	NCT02996461	NCT03110770
	VRC5288		prM-E	NCT02840487	
	GLS-5700	GeneOne Life Science, Inc./ Inovio Pharmaceuticals	prM-E	NCT02809443/ NCT02887482	
RNA	mRNA-1325	Moderna Therapeutics	prM-E	NCT03014089	
	mRNA-1893			NCT04064905	
Live Attenuated Viral Vectored	rZIKV/D4Δ30-713	NIAID	prM-E	NCT03611946	
	MV-ZIKV	Themis Bioscience GmbH	prM-sE	NCT02996890	
	MV-ZIKV-RSP		prM-E	NCT04033068	
	ChAdOx1 Zika	University of Oxford	prM-E	NCT04015648	
Inactivated Virus	ZPIV	NIAID/WRAIR/BIDMC	Whole virion	NCT02963909 NCT02952833 NCT02937233 NCT03008122	
	PZIV (TAK-246)	Takeda Pharmaceuticals	Whole virion	NCT03343626	
	BBV121	Bharat Biotech	Whole virion	CTRI/2017/05/ 008539	
	VLA1601	Valneva Austria GmbH/ Emergent Biosolutions	Whole virion	NCT03425149	

BIDMC, Beth Israel Deaconess Medical Center; ChAdOx1, Chimpanzee Adenovirus Oxford 1; NIAID, National Institute of Allergy and Infectious Diseases; PZIV, purified Zika inactivated vaccine; VRC, Vaccine Research Center; WRAIR, Walter Reed Army Institute of Research; ZPIV, ZIKV purified inactivated vaccine.
